# The safety and effectiveness comparison of Delta Medical's PEEK interface screw and Endobutton and that of Smith & Nephew's in arthroscopic anterior cruciate ligament reconstruction: A multicenter prospective double-blind randomized controlled clinical trial

**DOI:** 10.3389/fpubh.2022.1003591

**Published:** 2022-11-07

**Authors:** Peng Gao, Minghao Yuan, Yongsheng Xu, Yufeng Wu, Xiaohang Lin, Yanlin Li, Shensong Li, Jing Wang

**Affiliations:** ^1^Department of Joint Surgery and Sports Medicine, Hunan Provincial People's Hospital (The First-Affiliated Hospital of Hunan Normal University), Changsha, China; ^2^Clinical Research Center for Sports Medicine in Hunan Province, Changsha, China; ^3^Department of Bone and Joint Surgery, Inner Mongolia Autonomous Region People's Hospital, Huhhot, China; ^4^Department of Orthopedics, Traditional Chinese Medicine Hospital of Zhongshan, Zhongshan, China; ^5^Department of Sports Medicine, First Affiliated Hospital of Kunming Medical University, Kunming, China; ^6^Department of Sports Medicine, The 940 Hospital of Joint Logistics Support Force of Chinese People's Liberation Army, Lanzhou, China

**Keywords:** arthroscopic ACLR, PEEK interference screw, EndoButton, Lysholm knee scores, functional range of motion

## Abstract

**Background:**

To reduce the costs and financial burden in the ACLR treatment, we compare the early clinical outcomes and Magnetic Resonance Imaging (MRI) results of Delta Medical's PEEK (polyether ether ketone) interference screw and EndoButton with those of Smith & Nephew's PEEK interference screw and EndoButton in patients with arthroscopic anterior cruciate ligament reconstruction.

**Methods:**

A total of 104 patients in five different medical centers were randomly allocated into two groups: 1: Delta Medical's PEEK interference screw and EndoButton (53 patients); 2: Smith & Nephew's PEEK interference screw and EndoButton (51 patients). The modified Lysholm knee score, the laxity examination, and clinical and functional range of motion were evaluated at 3 and 6 months postoperatively. The clinical effective rate was calculated and classified as excellent and good at 6 months postoperatively. MRI examinations were performed at 3 and 6 months postoperatively to determine the healing process. Computerized tomography (CT) was performed at 2 weeks and 3 months postoperatively to evaluate the complications.

**Results:**

Significant improvements in knee function and functional scores were observed in both groups after surgery regardless of the fixation materials applied (*P* < 0.05). No differences were observed in the functional scores and range of motion. The assessments of Lysholm knee scores at 3 and 6 months produced no statistical differences (both *P* > 0.05). The clinical effective rate revealed no difference between the groups at 6 months postoperatively (non-inferiority analysis *P* = 0.0220). The differences of laxity examination between the groups were not statistically significant (Fisher's test, *P* = 0.6139, 0.2004, respectively). No significant differences in the functional range of motion were found at each follow-up time-point (*P* > 0.05). No major intra- or postoperative complications, such as infection, and vessel or nerve injury were observed.

**Conclusions:**

Knee function and functional scores were improved after ACLR in both groups, regardless of the PEEK interference screw and EndoButton applied. The difference in functional scores and range of motion were not significant in groups 1 and 2. Delta Medical's PEEK interference screw and EndoButton had a non-inferiority effect compared to Smith & Nephew's PEEK interference screw and EndoButton. Delta Medical's PEEK interference screw and EndoButton were suitable for arthroscopic ACLR.

## Introduction

Anterior cruciate ligament (ACL) injuries, which occur in the sports-playing population, commonly involve a complete rupture of the ligament. The incidence of ACL injuries rate has been steadily increasing and was most recently estimated at over 0.4% every year in adolescents (1). Surgical reconstruction of the ACL with arthroscopy has become the standard care procedure (2). It is reported that the annual incidence of ACL injuries patients who undergo the reconstructive procedure is nearing 300,000 in the United States (3). Normally, ACL injuries are treated by reconstructing the ruptured ligament with a graft, which can be taken from various sources including autograft, allograft, and artificial graft (4). The bone-patellar tendon-bone (BPTB) graft was applied as the “gold standard procedure” for many years. During the past two decades, the application of the semitendinosus and gracilis tendons as autografts for arthroscopic ACL reconstruction has increased dramatically (5). Compared with the use of BPTB graft, the benefits of using semitendinosus and gracilis tendons consist of less potential donor-site morbidity and lower influence on the extensor mechanism (6, 7).

Two main types of fixation devices are used in ACL reconstruction. One is aperture fixation like interference screw. The other is suspensory fixation like EndoButton^®^ (Smith & Nephew, Inc. Andover, MA, USA). Metallic interference screws (MISs) were the first applied interference screw that have reached a reliably positive clinical outcome (8). MISs can promote early bone integration with high initial fixation strength but a relatively high failure load which is very difficult to remove during revision surgery (9, 10). The recent improvements and developments in graft tendon fixation materials have contributed to the promising outcomes of ACL reconstruction procedures (11, 12). This further promotes the early rehabilitation process including range of motion and weight-bearing exercises. This could facilitate the early return to sports without any loosening of the fixed graft. Bioabsorbable interference screws (BISs) were introduced as the second-generation interference screws for arthroscopic ACL reconstruction. BISs can degrade in 2–3 years and simplify the revision surgery and minimized the subsequent MRI artifacts (13–15). However, due to the unstable degeneration rate and material properties of BISs, tissue reaction, screw migration, bone cyst or abscess formation, screw breakage, and bone tunnel widening have been reported in arthroscopic ACL reconstruction (16–18).

With the development of interference screw materials, polyether ether ketone (PEEK) has been introduced as a new interference screw material with the advantage of being chemically inert, insoluble, and radiolucent which has a closer elasticity modulus with cortical bone (19). There is no difference in tunnel widening or cyst formation compared with other commonly used materials for graft fixation (20). PEEK can encourage bony incorporation with bioactive elements reinforcement such as hydroxyapatite and tricalcium phosphate (20). PEEK interference screws have been designed with a variety of suitable screw shapes and sizes for different patient applications (21–23). Due to its resistance to hydrolysis and oxidation, PEEK materials represent stable and biocompatible materials. PEEK materials are very attractive for application in orthopedic surgery, which is considered to provide superior postoperative imaging and stable fixation benefits (21, 24, 25).

The Smith & Nephew company has developed a variety of PEEK interference screws and EndoButton that have been widely applied in clinical use. The clinical outcomes are promising and benefit patients significantly (26, 27). However, the financial burden for patients is relatively high, especially in developing countries with poor insurance coverage. To reduce the costs of the treatment, Delta Medical has developed a series of PEEK interference screws and Endobutton with a much lower cost, which has already been approved for the medical market by the National Medical Products Administration of China.

The purpose of this study was to compare the early clinical outcomes and MRI results of Delta Medical's PEEK interference screw and EndoButton with those of Smith & Nephew's PEEK interference screw and EndoButton in patients with arthroscopic ACL reconstruction. The hypothesis is that the Delta Medical's PEEK interference screw and EndoButton have a non-inferiority effect in patients with arthroscopic ACL reconstruction compared with those of Smith & Nephew's PEEK interference screw and EndoButton.

## Materials and methods

### Sample size calculation

In this study, we aimed to prove that the clinical effects of the Delta Medical's PEEK interface screw and Endobutton products were not inferior to the similar Smith & Nephew's PEEK interface screw and Endobutton in arthroscopic ACL reconstruction. The sample size was determined according to the non-inferiority test. The qualitative index (effective rate) was adopted as an evaluation index.


$$\begin{array}{l} n=n_{1}=n_{2}=\frac{2\times {\left(t_{\alpha }+t_{\beta }\right)}^{2}P\;\left(1-P\right)}{\delta^{2}}\; \end{array}$$

The significance level α value, power 1–β, non-inferiority threshold δ, and average total effective rate P value were determined according to the actual investigation of the efficacy of this type of product and the general statistical requirements. Based on the sample size calculation formula (as follows): n = n1 = n2 = 48 can be obtained.

Considering the possibility of cases falling off, the sample size of each group was set as 53 cases; that is, 53 cases in the experimental group and 53 cases in the control group.

n: estimated sample size; n1 and n2 are the sample sizes of the experimental and control groups, respectively.

P: the average effective rate of the control group. Combined with the actual work experience of the main investigator, the average effective rate of the positive control substance P was conservatively determined as 97%.

δ: non-inferiority cut-off value. According to the “Guiding Principles for Selection of Non-inferiority Cut-off Value” issued by the European Drug Evaluation Organization (EMEA) (EMEA/CPMP/EWP/2158/99), and by the International Coordination Conference on Technical Requirements for Registration of Human Drugs (ICH) E9, E10 guidelines, a value of 0.1 was determined as the non-inferiority threshold, which was close to the10% (0.097) average effective rate of the control group.

α: significance level (false positive rate). According to “Biostatistics Technical Guidelines for Clinical Trials of Chemical Drugs and Biological Products”, α was set as 0.05 on both sides.

β: 1–β is the test efficiency (power). According to the “Biostatistics Technical Guidelines for Clinical Trials of Chemical Drugs and Biological Products”, 1–β was set as 0.8.

### Study design and patient selection

This study was a multicenter (Hunan Provincial People's Hospital, The First Affiliated Hospital of Hunan Normal University, People's Hospital of Inner Mongolia Autonomous Region, Zhongshan Traditional Chinese Medicine Hospital, The First Affiliated Hospital of Kunming Medical University, The 940th Hospital of Joint Logistics Support Force of The Chinese People's Liberation Army), prospective, double-blind, randomized, controlled, clinical trial. A total of 104 consecutive patients who underwent arthroscopic ACLR between November 1, 2017, and February 27, 2020, were enrolled in five different medical centers and randomly divided into two groups. Group 1 used Delta Medical's PEEK interference screw and EndoButton, while group 2 used Smith & Nephew's PEEK interference screw and EndoButton.

Block randomization was performed to allocate patients to one of the two treatment groups. An independent investigator, who was not involved in the surgical treatment, prepared and sealed opaque envelopes bearing the type of PEEK interference screw and EndoButton used. Following diagnostic arthroscopy, the patients were randomized into one of the two treatment groups. Patients were not informed about which material was used, either on the day of the surgery or at the follow-up visits. Also, the examiners who evaluated the patients' knees did not know the material used.

Patients were enrolled in this study when an ACL injury was diagnosed based on clinical examination and an MRI test. All procedures performed in this study involving human participants were in accordance with the Declaration of Helsinki (as revised in 2013). The study was approved by the medical ethics board of Hunan Provincial People's Hospital, The First Affiliated Hospital of Hunan Normal University (NO. 2017-06.1), People's Hospital of Inner Mongolia Autonomous Region (NO.YWLCSYLL-2017-004-01), Zhongshan Traditional Chinese Medicine Hospital (NO.2017ZSZY-LL-002), The First Affiliated Hospital of Kunming Medical University (NO.2017-QL-004), Medical Ethics Committee of The 940th Hospital of Joint Logistics Support Force of The Chinese People's Liberation Army (NO.2018-QL-002), separately. The informed consent was taken from all the patients.

The inclusion criteria were as follows:

(I) Patients have signed the informed consent; (II) Patients aged 18 to 65 (including 18 and 65 years old), regardless of gender; (III) Patients meet the diagnosis of anterior cruciate ligament rupture which requires arthroscopic reconstruction of the anterior cruciate ligament, and have no contraindications to implantation; (IV) Patients have good compliance, and willing to conduct follow-up observation as required.

The exclusion criteria were as follows:

(I) Patients who have participated in other medical device trials within 3 months; (II) Patients with severe allergies; (III) Patients with abnormal liver and kidney function and coagulation disorders; (IV) Patients who have serious heart and lung diseases that restrict their participation in the study; (V) Patients have poor compliance with mental disorder; (VI) Patients have a positive pregnancy test; (VII)There is evidence that the subject abuses drugs; (VIII)Patients with peripheral nerve injury at the surgical site; (IX) Patients with myocardial infarction within 6 months and a history of cerebral infarction within 3 months; (X)The investigator believes that there are other circumstances that patients are inappropriate to participate in this clinical trial.

### Harvest and preparation of grafts

**Figure 1:**
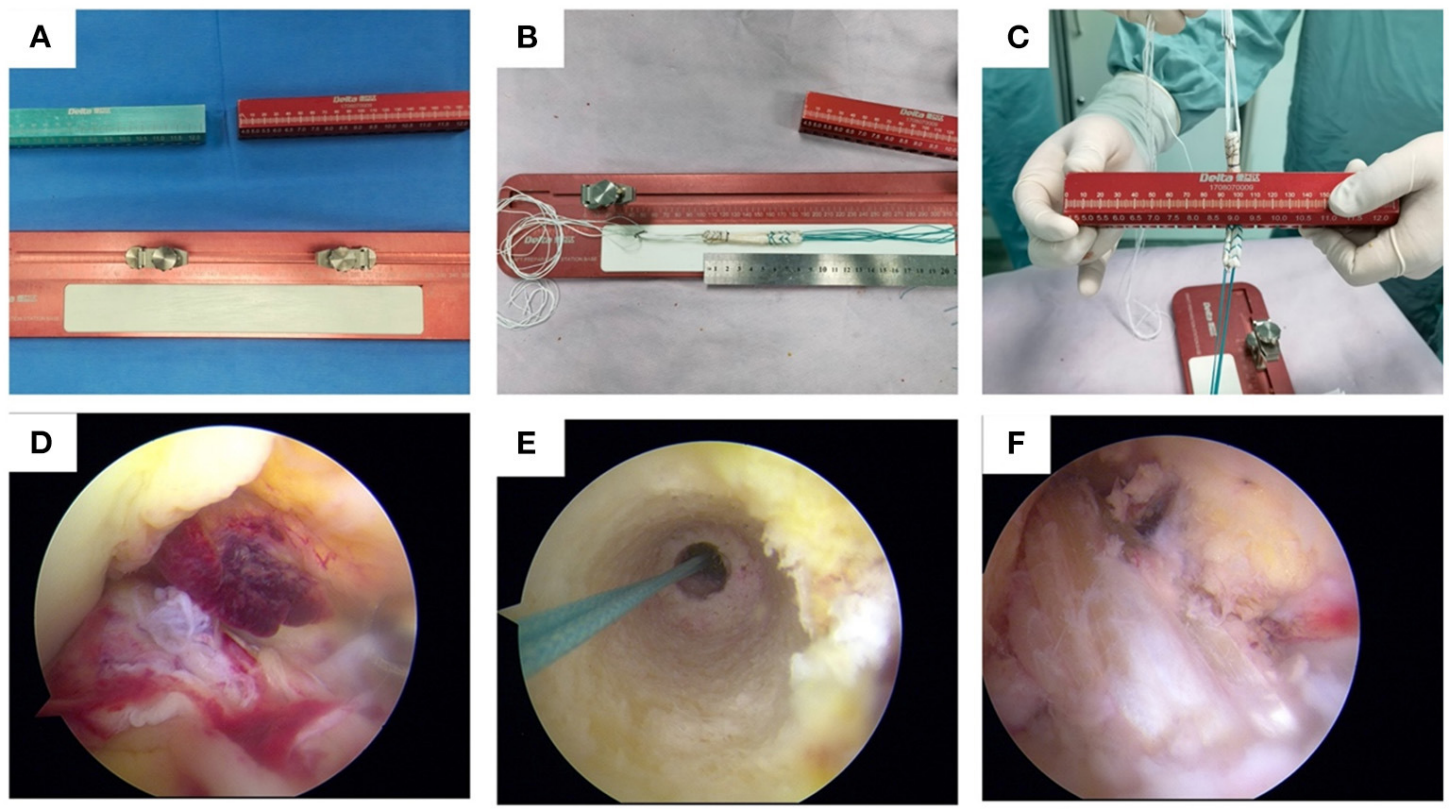
Surgical procedures of ACL reconstruction. **(A)** the tendon preparation panel of Delta Medical; **(B)** measuring the length of the autogenous tendon; **(C)** measuring the diameter of the autogenous tendon; **(D)** confirming the diagnosis of ACL injury; **(E)** preparing the femoral tunnel; **(F)** final repair configuration of the ACL reconstruction.

General anesthesia was administered to all patients while in the supine position. The injured knee was placed with an unsterile tourniquet around the upper thigh which allowed greater than 120 degree of knee flexion. The semitendinosus and gracilis tendons were harvested with a tendon stripper (Delta Medical). The residual soft tissue was cleaned from the tendons. The distal free ends of the tendons were armed with No. 6 Ethibond sutures using a whipstitch technique. Then the semitendinosus and gracilis tendons were folded in half and looped over Delta Medical's EndoButton in group 1 and Smith & Nephew's EndoButton in group 2. The diameter of the graft was ~7.0–9.0 mm. All the grafts were pretensioned under 20 pounds for 20 min and marked with absorbable suture 2 cm at both ends (Figures 1A–C).

### Surgical technique

**Figure 2:**
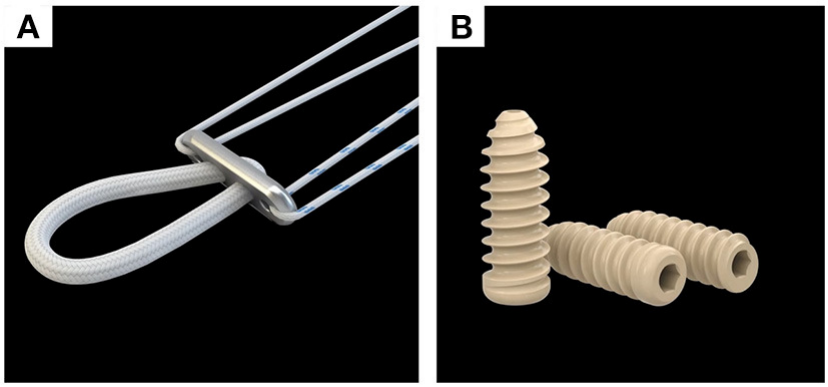
Endobutton **(A)** and PEEK interface screw **(B)** of Delta Medical applied in group 1.

Arthroscopic ACLR surgeries were performed by five senior orthopedic surgeons in the five different medical centers (i.e., the same senior orthopedic surgeon in each medical center). The surgeon who performed the surgery did not do the follow-up. A 3-portal technique was used with a anterolateral portal, anteromedial portal, and lower accessory anteromedial portal. Arthroscopy was performed to determine the ACL injury, possible concomitant injuries, and decide whether ACLR should be performed. Before the reconstruction, the meniscal lesion was managed. Debridement or microfracture was done to the chondral damages. Once the ACL injury had been defined, the tibial and femoral tunnel were marked and prepared. The sizes of the tunnels were drilled according to the precise diameter of the graft (Figures 1D–F). After the femoral and tibial tunnels were prepared, the graft-button complex was passed through the tunnel first and the button (Figure 2A) was flipped over the lateral cortex of the femur to secure the fixation. Then the tibial side grafts were firmly pulled and fixed with Delta Medical's PEEK interference screw (Figure 2B) in group 1 and Smith & Nephew's PEEK interference screw in group 2 which had the same diameter as the drilled tunnel. Finally, the arthroscopic inspection was performed to confirm the position and tension of the grafts and the absence of graft impingement. After surgery, the knee was placed in extension with a brace.

### Rehabilitation

The two groups received the same postoperative management and rehabilitation. Before surgery, physical therapy was applied to restore full knee ROM and eliminate knee swelling. After surgery, active straight leg raise was initiated to strengthen the quadriceps immediately. ROM exercises started on the second day to obtain the full extension as compared with the contralateral side. Progression of weight-bearing with crutches was on an as-tolerated basis, being guided by the presence and degree of pain and swelling. Proprioception activities start at 8 weeks postoperatively and extend through ~4–6 months. Contact sports were not allowed until at least 6 months after surgery. For patients with meniscal and chondral injury, appropriate modifications to the ROM limits and weight-bearing status were made.

### Clinical evaluations

The clinical evaluation of the involved knee was performed at each medical center by one independent physical therapist who was not involved in the surgical repair process. The objective and subjective outcomes of the ACL reconstruction were obtained preoperatively and at each follow-up point.

The modified Lysholm knee scoring scale was applied to evaluate knee pain (25 points), degree of instability (25 points), locking sensation (15 points), swelling (10 points), using cane or crutches (5 points), limp (5 points), climbing stairs (10 points), and squatting (5 points). The maximum obtainable score is 100 points. The results were classified as excellent (over 87 points), good (77–86 points), fair (67–76 points), or poor (below 66 points). The clinical effective rate was calculated based on the Lysholm knee scoring results and classified as excellent and good at 6 months postoperatively.

The laxity examination of the involved knee after ACL reconstruction included the Lachman test and Drawer test. Clinical and functional range of motion (both active and passive) evaluations were performed on all patients preoperatively, and at 3 and 6 months postoperatively. Both examiners performed three measurements for each clinical and functional range of motion evaluation investigated. The average value for each variable was used for statistical analysis.

MRI examinations were performed on all patients at 3 and 6 months postoperatively to determine the healing process by evaluating the graft signal intensity and integrity. Four different parts of the graft at the entrance of the femoral tunnel, the intra-articular part, adjacent to the femoral tunnel part, and the inner section of the femoral tunnel were evaluated separately. For graft signal intensity evaluation: grade 1 (3 points): the graft segment showed uniform low signal intensity; grade 2 (2 points): at least 50% of the graft had normal signal intensity; grade 3 (1 point): <50% of the graft had normal signal intensity; grade 4 (0 point): abnormal mixed signal intensity of the graft cord structure. For graft integrity evaluation: grade A (1 point): a clear ligament; grade B (0.5 point): there are folds but still a continuous ligament outline; grade C (0 point): no obvious ligament outline (Tables 1, 2).

**Table 1:** The MRI graft signal intensity scale.

	**Grade 1 (3 point)**	**Grade 2 (2 point)**	**Grade 3 (1 point)**	**Grade 4 (0 points)**
The entrance of the femoral tunnel	□	□	□	□
The intra-articular part	□	□	□	□
Adjacent to the femoral tunnel part	□	□	□	□
The inner section of the femoral tunnel	□	□	□	□
Graft signal intensity score	

Grade 1 (3 points): the graft segment showed uniform low signal intensity; grade 2 (2 points): at least 50% of the graft had normal signal intensity; grade 3 (1 point): <50% of the graft had normal signal intensity; grade 4 (0 points): abnormal mixed signal intensity of the graft cord structure.

**Table 2:** The MRI graft signal integrity scale.

Grade A, a clear ligament	1 point □
Grade B, folds but still a continuous ligament outline	0.5 point □
Grade C, no obvious ligament outline	0 point □

Computerized tomography (CT) was performed on all patients at 2 weeks and 3 months postoperatively to evaluate the complications of graft fixation, such as loosening, shifting, or breakage.

### Statistical analysis

We used SAS (version 9.4; Statistical Analysis System) for statistical analysis. Data are presented as percentages for categorical variables, and as means and standard deviations for continuous variables. The statistical description was performed on demographic characteristics. Continuous variables were described using the mean, standard deviation, median, minimum, and maximum values. Categorical variables were described using frequencies. Baseline equilibrium statistical inferences were made on the demographic characteristics. Continuous variables used two independent samples *t*-test or Wilcoxon rank-sum test according to the situation. Categorical variables used two independent samples chi-square test or Fisher exact probability method according to the situation. A Per-Protocol Set (PPS) was used to conduct statistical analysis of the trial efficacy. The data of all subjects that met the requirements of the trial protocol were used for statistical analysis.

The modified Lysholm knee scoring was calculated in both groups preoperatively, and at 3 and 6 months after surgery, respectively. Two independent samples *t*-test or Wilcoxon rank-sum test were applied to analyze the differences between the two groups. The clinical effective rate was calculated according to the modified Lysholm knee scoring scales results at 6 months postoperatively, and the 95% confidence interval of the clinical effective rate difference between the two groups was finally calculated.

Concerning the Lachman test, drawer test, and CT scanning results, two independent samples chi-square test or Fisher exact probability method were used for categorical variables. For clinical and functional range of motion (both active and passive) evaluations and MRI examination, two independent samples *t*-test or Wilcoxon rank-sum test were used for continuous variables. Statistical significance was defined as *P* < 0.05.

## Results

**Figure 3:**
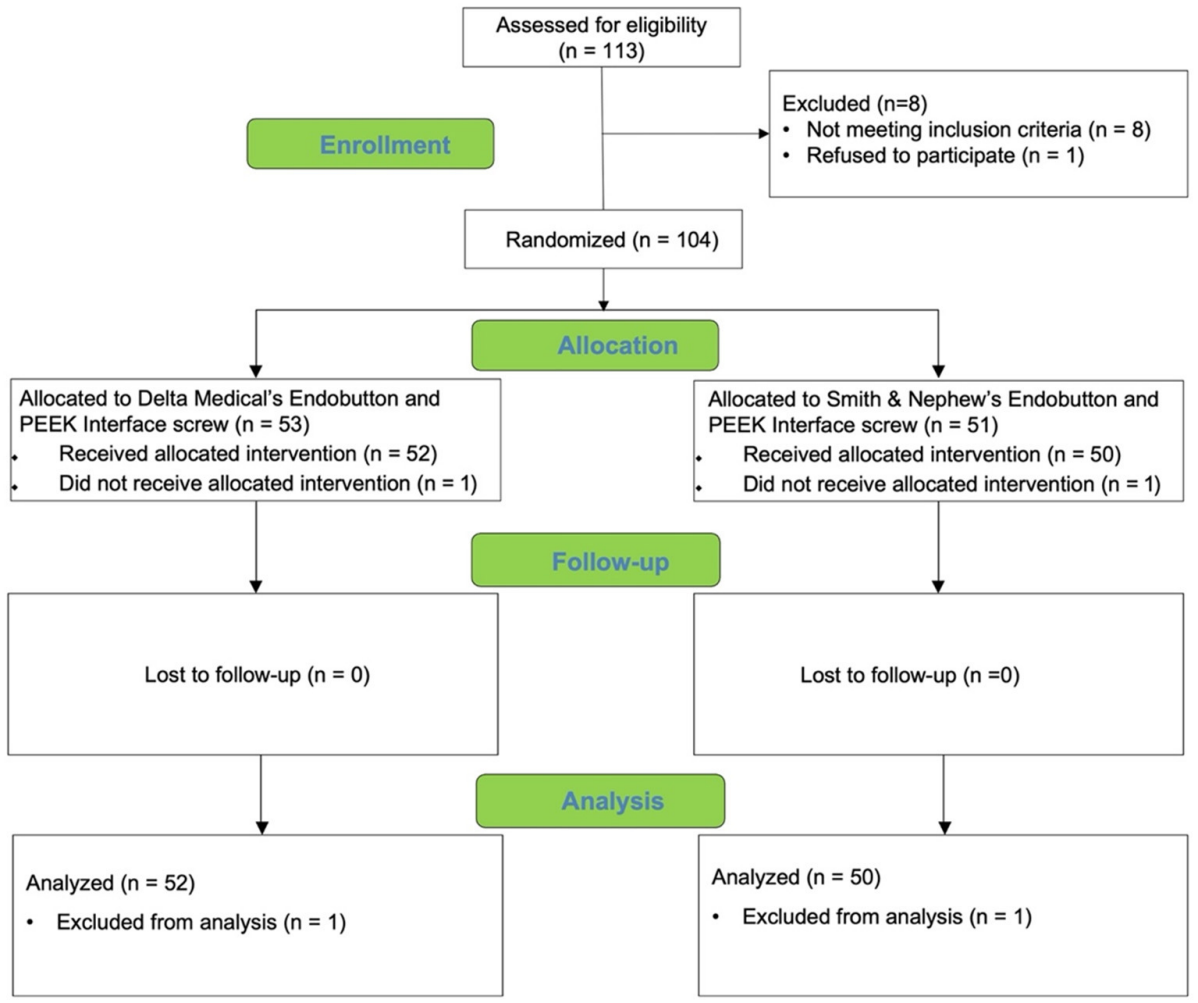
CONSORT (Consolidated Standards of Reporting Trials) flow diagram.

A total of 104 patients were enrolled in this study (53 patients in group 1 and 51 patients in group 2). One patient in group 1 was excluded during hospitalization because of the exclusion criteria. One patient in group 2 planned to use the experiment interface screw which was contaminated during the operation. To ensure the safety of the patient, another interface screw from another company was applied. We still did the full follow-up for these two patients but excluded them from the trial analysis. So a total of 102 patients' data were analyzed in this study. All patients completed the follow-up (Figure 3). The mean age of the patients was 32.5 years (range, 18–65 years), and they all had a follow-up time of 6 months. The study population consisted of 71 (68.3%) men and 33 (31.7%) women. The mean height of the studied population was 169 cm (range, 150–190 cm), with a standard deviation of 8 cm. The mean bodyweight of the studied population was 70.36 kg (range, 45–100 kg), with a standard deviation of 12.95 kg (Table 3).

**Table 3:** Demographics data of the two groups.

**Variables**	**Group 1**	**Group 2**	***P-*value**
Number of cases	53	51	
Median age (SD)	32.5 (11.15)	33.0 (11.76)	0.6423
Male/female	37/16	34/17	0.7305
Height (SD)	1.69 (0.086)	1.70 (0.074)	0.9230
Weight (SD)	69.62 (13.121)	71.14 (12.861)	0.5505

The *P*-value is derived from the t-test or chi-square test. SD: standard deviation.

### The modified Lysholm knee scoring scale

Before the operation, the mean (SD) Lysholm knee scores were 52.7 points (21.46) and 51.8 points (27.26) in groups 1 and 2, respectively, and there was no statistically significant difference between the two groups (*P* = 0.8480). At 3 and 6 months after surgery, patients in both groups had a significant improvement in their Lysholm knee scores. The mean (SD) Lysholm knee scores in groups 1 and 2 were 80.5 points (14.85) and 83.4 points (11.60) at 3 months, respectively, compared with that 52.7 points (21.46) and 51.8 points (27.26) in groups 1 and 2, preoperatively (both *P* < 0.05). At 6 months post-operation, the mean (SD) Lysholm knee scores in groups 1 and 2 were 87.9 points (11.04) and 89.2 points (13.62), respectively. Neither of the assessments of Lysholm knee scores between the two groups at 3 and 6 months postoperatively showed statistically significant differences (Fisher's test, *P* = 0.2693 and 0.6203, respectively) (Table 4). Furthermore, knee pain, degree of instability, locking sensation, swelling, using canes or crutches, limp, climbing stairs, and squatting, respectively, neither of these parameters showed statistically significant differences between the two groups throughout the entire follow-up period.

**Table 4:** Functional assessment: The modified Lysholm knee scoring scale.

**Groups**	***N***	**Mean (SD)**	***P-*value**
**Preoperatively**			0.8400
Group 1	52	52.7 (21.46)	
Group 2	50	51.8 (27.26)	
**3 months**			0.2693
Group 1	52	80.5 (14.85)	
Group 2	50	83.4 (11.60)	
**6 months**			0.6203
Group 1	52	87.9 (11.04)	
Group 2	50	89.2 (13.62)	

The *P*-value is derived from Fisher's test. SD: standard deviation.

### The clinical effective rate

The clinical effective rate was calculated based on the Lysholm knee scoring results and classified as excellent or good at 6 months postoperatively. After 6 months of surgery, five patients in group 1 did not achieve excellent or good Lysholm knee scores, while in group 2, six patients did not obtain excellent or good Lysholm knee scores, which was slightly higher than group 1. Based on the Lysholm knee scoring results at 6 months postoperatively, the clinical effective rate in group 1 was 90.4%, compared with 88.0% in group 2. However, the difference between the groups was not statistically significant (non-inferiority analysis *P* = 0.0220). The 95% confidence interval was −0.0967 to 0.1444 (Table 5).

**Table 5:** Clinical effective rate^a^.

**Groups**	**Excellent or good**	**Rate**	**Difference test**		**Non-inferiority analysis**		**95% CI**	
			**χ^2^**	***P-*value**	**Z**	***P-*value**	**Upper**	**Down**
Group 1	47	90.4%	0.1507	0.6979	2.0135	0.0220	−0.0967	0.1444
Group 2	44	88.0%						

^a^This data is based on the Lysholm knee score. CI: confidence interval.

### The laxity examination

The laxity examination of the involved knee after ACL reconstruction included the Lachman test and Drawer test. Before surgery, 51 of 52 patients in group 1 and 49 of 50 patients in group 2 showed a positive sign in the pre-Lachman Test, and 51 of 52 patients in group 1 and 48 of 50 patients in group 2 showed a negative sign in the post-Lachman test. No significant difference was observed between these two groups. After 3 months of surgery, there were 51 of 52 (98.1%) patients in group 1 and 48 of 50 (96.0%) patients in group 2 had a negative sign in the pre-Lachman Test which mean the reconstructed ACL had provided enough strength support for the stability of the involved knee. The same results were observed in these two groups at 6 months post-operation. Meanwhile, the differences between the groups were not statistically significant (Fisher's test, *P* = 0.6139, 0.2004, respectively) (Table 6).

**Table 6:** Lachman test results.

**Variable**	**Preoperatively**			**3 months**			**6 months**		
	**Group 1**	**Group 2**	***P-*value**	**Group 1**	**Group 2**	***P-*value**	**Group 1**	**Group 2**	***P-*value**
Positive pre-Lachman test	51 (98.1%)	49 (98.0%)	1.0000	1 (1.9%)	2 (4.0%)	0.6139	1 (1.9%)	4 (8.0%)	0.2004
Negative pre-Lachman test	1 (1.9%)	1 (1.9%)		51 (98.1%)	48 (96.0%)		51 (98.1%)	46 (92.0%)	
Positive post-Lachman test	1 (1.9%)	2 (4.0%)	0.6139	0	0	/	1 (1.9%)	2 (4.0%)	0.6139
Negative post-Lachman test	51 (98.1%)	48 (96.0%)		52 (100.0%)	50 (100.0%)		51 (98.1%)	48 (96.0%)	

The *P*-value is derived from Fisher's test.

There were 50 of 52 patients in group 1 and 50 of 50 patients in group 2 showed a positive sign in the pre-drawer Test, and all the patients in group 1 and group 2 showed a negative sign in the post- drawer Test before surgery. After 3 months of surgery, 50 of 52 (96.2%) patients in group 1 and 48 of 50 (96.0%) patients in group 2 had a negative sign in the pre-drawer Test. Both groups had two patients who showed a negative sign in the pre-drawer Test. There were 49 of 52 (94.2%) patients in group 1 and 46 of 50 (92.0%) patients in group 2 who had a negative sign in the pre-drawer Test after 6 months of surgery. No significant differences were observed among these two groups at each time point (Fisher's test, *P* = 0.4954, 1.0000, 0.7127, respectively) (Table 7).

**Table 7:** Drawer test results.

**Variable**	**Preoperatively**			**3 months**			**6 months**		
	**Group 1**	**Group 2**	***P-*value**	**Group 1**	**Group 2**	***P-*value**	**Group 1**	**Group 2**	***P-*value**
Positive pre-drawer test	50 (96.2%)	50 (100.0%)	0.4954	2 (3.8%)	2 (4.0%)	1.0000	3 (5.8%)	4 (8.0%)	0.7127
Negative pre-drawer test	2 (3.8%)	0		50 (96.2%)	48 (96.0%)		49 (94.2%)	46 (92.0%)	
Positive post- drawer test	0	0	/	0	0	/	2 (3.8%)	2 (4.0%)	1.0000
Negative post- drawer test	52 (100.0%)	50 (100.0%)		52 (100.0%)	50 (100.0%)		50 (96.2%)	48 (96.0%)	

The *P*-value is derived from Fisher's test.

### Clinical and functional range of motion measurement

Clinical and functional range of motion (both active and passive) evaluations were performed on all patients preoperatively, and at 3 and 6 months postoperatively. The mean active range of flexion motion increased significantly in both groups after ACL reconstruction. In group 1, the mean active flexion range of motion improved from 117.4 degrees preoperatively to 137.3 degrees at 6 months postoperatively (*P* < 0.05), while the mean passive flexion range of motion improved from 124.0 degrees preoperatively to 139.7 degrees at 6 months postoperatively (*P* < 0.05). In group 2, the mean active range of flexion motion improved from 119.8 degrees pre-operatively to 137.7 degrees at 6 months postoperatively (*P* < 0.05), while the mean passive flexion range of motion improved from 126.1 degrees preoperatively to 139.9 degrees at 6 months postoperatively (*P* < 0.05). No significant differences in the range of flexion motion were found at each follow-up time-point between the two groups (both *P* > 0.05). Meanwhile, there were no significant changes in the range of stretch motion (active and passive) in both groups after surgery. The mean active range of stretch motion in group 1 was 1.0 before surgery, compared with 0.0 at 6 months after surgery (*P* > 0.05). The mean range of stretch motion in group 2 was −0.4 before surgery, compared with 0.1 at 6 months after surgery (*P* > 0.05) (Table 8).

**Table 8:** Functional range of motion measurement.

**Variable**	**Preoperatively**			**3 months**			**6 months**		
	**Group 1**	**Group 2**	***P-*value**	**Group 1**	**Group 2**	***P-*value**	**Group 1**	**Group 2**	***P-*value**
Stretch (active activity)	1.0 (5.78)	−0.4 (6.02)	0.1789	−0.1 (2.52)	0.7 (2.22)	0.0461	0.0 (1.28)	0.1 (1.88)	0.5416
Stretch (passive activity)	0.7 (4.09)	0.1 (5.05)	0.5215	2.1 (14.06)	0.7 (2.44)	0.5023	−0.1 (1.46)	0.3 (1.99)	0.1933
Flexion (active activity)	117.4 (24.20)	119.8 (23.25)	0.5587	129.5 (14.43)	129.6 (15.95)	0.9568	137.3 (12.31)	137.7 (12.50)	0.9566
Flexion (passive activity)	124.0 (22.73)	126.1 (19.72)	0.6839	134.9 (11.90)	134.8 (14.14)	0.9622	139.7 (11.38)	139.9 (13.11)	0.8765

The *P*-value is derived from t-test or non-parametric test.

### MRI examination results

**Figure 4:**
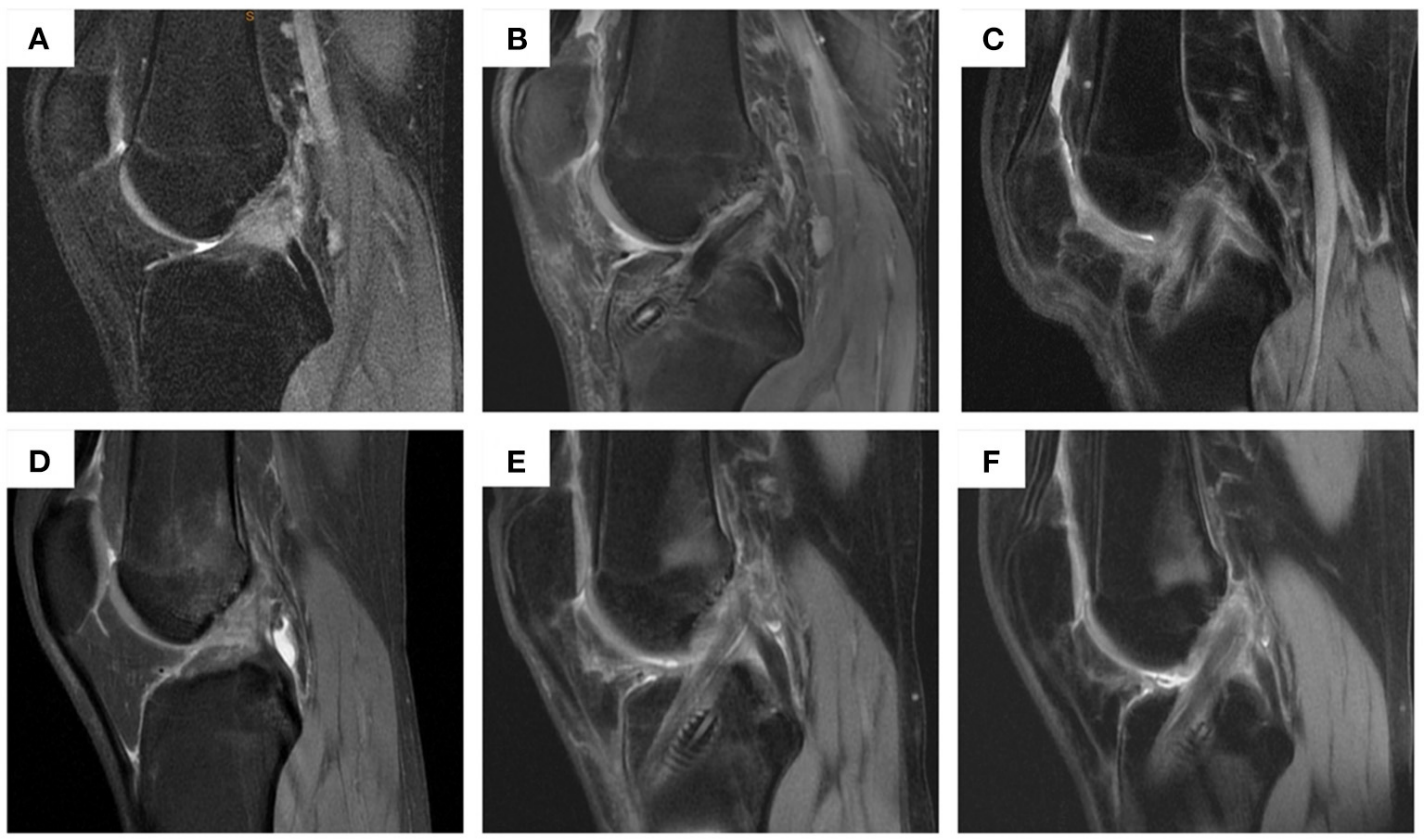
MRI examination of patients in group 1 and group 2 before operation and at 3 and 6 months post-operation. **(A)** patients in group 1 before operation; **(B)** patients in group 1 at 3 months post-operation; **(C)** patients in group 1 at 6 months post-operation; **(D)** patients in group 2 before operation; **(E)** patients in group 2 at 3 months post-operation; **(F)** patients in group 2 at 6 months post-operation.

MRI examinations were performed on all patients at 3 and 6 months postoperatively to determine the healing process by evaluating the graft signal intensity and integrity (Figure 4). The graft Strength and Integrity score was measured based on MRI results. The mean score in group 1 was 11.5 points and 11.0 points in group 2 after 3 months of ACL reconstruction. After 6 months of surgery, the mean score in group 1 was 11.0 points and 10.7 points in group 2. No significant differences were observed among these two groups at 3 months and 6 months of ACL reconstruction (Fisher's test, *P* = 0.0898 and 0.4567, respectively) (Table 9).

**Table 9:** Graft Strength and Integrity Score based on MRI.

**Time**	**Result**	**Group1**	**Group 2**	***P-*value**
3 months	*N* (Nmiss)	52 (0)	50 (0)	0.0898
	Mean (SD)	11.5 (2.36)	11.0 (2.26)	
	Median	12.5	12.0	
	Q1, Q3	11.0,13.0	10.0,13.0	
	Min–Max	0–13	5–13	
6 months	*N* (Nmiss)	52 (0)	50 (0)	0.4567
	Mean (SD)	11.0 (2.86)	10.7 (2.64)	
	Median	12.0	12.0	
	Q1,Q3	9.8,13.0	9.0,13.0	
	Min–Max	0–13	4–13	

The *P*-value is derived from t-test or Wilcoxon rank-sum test.

### Complications

**Figure 5:**
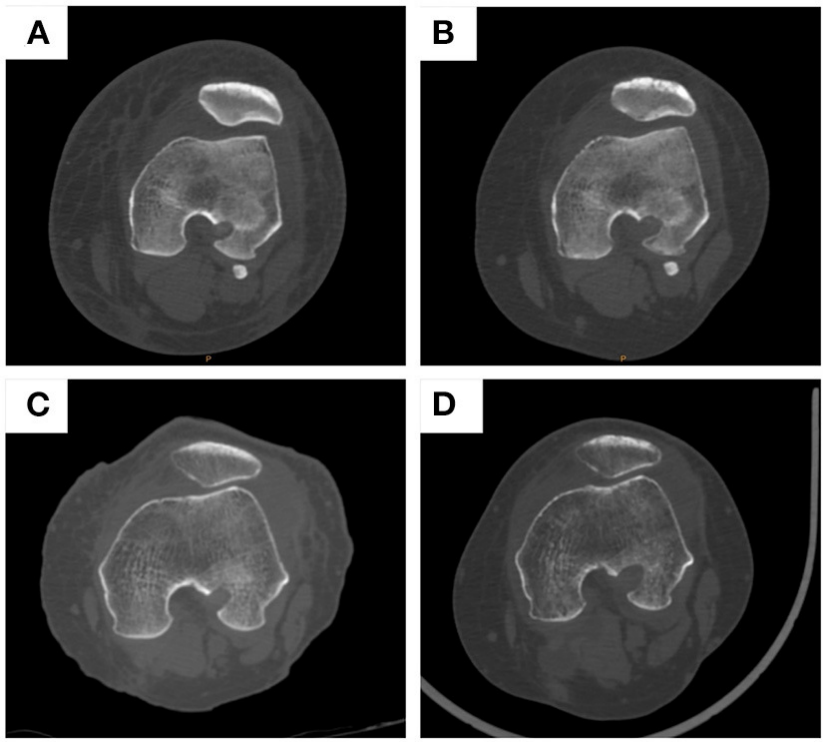
CT examination of patients in group 1 and group 2 at 2 weeks and 3 months post-operation. **(A)** patients in group 1 at 2 weeks post-operation; **(B)** patients in group 1 at 3 months post-operation; **(C)** patients in group 2 at 2 weeks post-operation; **(D)** patients in group 2 at 3 months post-operation.

CT was performed on all patients at 2 weeks and 3 months postoperatively to test the complications, such as loosening, shifting, or breakage. Based on the results, there was only one patient in group 2 who had a screw loosening at 3 months postoperatively. No shifting or breakage occurred in both groups at 2 weeks and 3 months postoperatively (Figure 5). There were no major intra- or postoperative complications, such as infection, and vessel or nerve injury. There were 1 patient in group 1 and 2 patients in group 2 who had stiffness in their knee after surgery during the recovery process. All stiffness in both groups improved significantly after active function exercise.

## Discussion

Arthroscopic ACL reconstruction with graft is considered the standard procedure for ACL injuries. The hamstring tendons were most frequently used for graft selection. Though there is no consensus on the choice of graft fixation devices for ACLR, most surgeons used interference screw for tibial fixation and suspension device for femoral fixation. In the UK, 79% of hamstring tendons graft femoral fixation was done with a suspension device, and 18% was done with an interference screw (28). The EndoButton was most common (48%) applied as a suspension device. Some researchers concluded that the type of graft fixation device did not affect the clinical outcome and stability (29).

In this study, we investigated the PEEK interference screw and EndoButton developed by Delta Medical in arthroscopic ACL reconstruction and compared its clinical and MRI outcomes with those of Smith & Nephew's PEEK interference screw and EndoButton. Considering the modified Lysholm knee scoring scale, there were 47 patients in group 1 (90.4%) who achieve excellent or good Lysholm knee scores, while in group 2, there were 44 patients (88.0%) who obtain excellent or good Lysholm knee scores. However, the difference between the groups was not statistically significant. At 6 months post-operation, the mean (SD) Lysholm knee scores in groups 1 and 2 were 87.9 points (11.04) and 89.2 points (13.62), respectively. Guglielmetti reported the same result in his ACL reconstruction using metal interference screws. The mean Lysholm score of the patellar tendon group was 89.87 points (range, 65-100), and that of the hamstring tendon group was 91.26 points (range, 60-100) at 2 years post-operation (30).

The laxity examination of the involved knee after ACL reconstruction was performed to measure the graft strength and knee stability. After 3 months of surgery, there were 51 of 52 (98.1%) patients in group 1 and 48 of 50 (96.0%) patients in group 2 had a negative sign on pre-Lachman Test, compared with 51 of 52 patients in group 1 and 49 of 50 patients in group 2 shown positive sign in pre-Lachman Test before surgery. A systematic review and meta-analysis showed that 926 patients were evaluated for Lachman test grade 0 in 14 studies and negative Lachman test (grade 0) was found in 81.2% of cases (95% CI, 78.7–83.7%). Moreover, 14 studies evaluated 1029 patients for Lachman test grade 0 or 1, which was found in 96.1% of cases (95% CI, 94.9– 97.3%) (7). Similar results were found in the pre-drawer Test which means the reconstructed ACL had provided enough strength support for the stability of the involved knee.

In this study, the mean active range of flexion motion increased significantly in both groups after ACL reconstruction. Harris reported in his study that there was no loss of extension >3 degrees as compared with the contralateral knee in any patient. There was no loss of flexion >5 degrees as compared with the contralateral knee in any ACL reconstruction patient who completed objective follow-up (31). The mean active flexion range of motion in this study improved from 117.4 degrees preoperatively to 137.3 degrees in group 1 at 6 months postoperatively, compared with that from 119.8 degrees preoperatively to 137.7 degrees in group 2. No significant differences in the range of flexion motion were found at each follow-up time-point between the two groups.

MRI examinations were performed on all patients at 3 and 6 months postoperatively to determine the healing process. There were no significant differences between these two groups. No shifting or breakage occurred in both groups at 2 weeks and 3 months postoperatively based on CT examination. There were no major intra- or postoperative complications, such as infection, and vessel or nerve injury.

This multicenter, prospective, double-blind, randomized, controlled, clinical trial revealed that ACL injured patients repaired using PEEK interference screw and EndoButton developed either by Delta Medical or Smith & Nephew under arthroscopy had a significant improvement in clinical outcomes after surgery. When comparing Delta Medical's PEEK interference screw and EndoButton with Smith & Nephew's PEEK interference screw and EndoButton, no significant differences were observed in terms of modified Lysholm knee scoring, the clinical effective rate, the laxity examination, and clinical and functional range of motion. This indicates that Delta Medical's PEEK interference screw and EndoButton have a non-inferiority effect compared with Smith & Nephew's PEEK interference screw and EndoButton. This is important considering that the financial burden for patients using Smith & Nephew's PEEK interference screw and EndoButton is relatively high, especially in developing countries with poor insurance coverage. Delta Medical's products with a non-inferiority effect and much lower cost, which have already been approved for the medical market by the National Medical Products Administration of China, maybe a suitable alternative for ACL injured patients, especially those with lower incomes. The limitation of this trial is that the follow-up time was relatively short, and a longer follow-up time is needed to verify the long-term effect of Delta Medical's PEEK interference screw and EndoButton.

## Conclusion

Knee function and functional scores were improved after complete anterior cruciate ligament reconstruction in both groups, regardless of the PEEK interference screw and EndoButton applied. The difference in functional scores and range of motion were not significant in groups 1 and 2. Delta Medical's PEEK interference screw and EndoButton had a non-inferiority effect compared to Smith & Nephew's PEEK interference screw and EndoButton. Delta Medical's PEEK interference screw and EndoButton were suitable for arthroscopic anterior cruciate ligament reconstruction.

## Data availability statement

The raw data supporting the conclusions of this article will be made available by the authors, without undue reservation.

## Ethics statement

The study was approved by the Medical Ethics Board of Hunan Provincial People's Hospital, The First Affiliated Hospital of Hunan Normal University (No. 2017-06.1), People's Hospital of Inner Mongolia Autonomous Region (No. YWLCSYLL-2017-004-01), Zhongshan Traditional Chinese Medicine Hospital (No. 2017ZSZY-LL-002), The First Affiliated Hospital of Kunming Medical University (No. 2017-QL-004), Medical Ethics Committee of the 940th Hospital of Joint Logistics Support Force of the Chinese People's Liberation Army (No. 2018-QL-002), separately. The informed consent was taken from all the patients. The patients/participants provided their written informed consent to participate in this study.

## Author contributions

Conception and design: PG and JW. Administrative support: YX and JW. Provision of study materials or patients: YL, YW, and XL. Collection and assembly of data: MY, YX, YL, and YW. Data analysis and interpretation: PG and SL. Manuscript writing and final approval of manuscript: All authors. All authors contributed to the article and approved the submitted version.

## Funding

This work was supported, in part, by the Scientific Research Project of Hunan Provincial Health Special Fund (A2020-02, PG), 2022 Annual Scientific Research Project of Hunan Provincial Health Commission (202204073112, PG, 202204073117, JW), 2021 Hunan Provincial Department of Education Scientific Research Project (21B0041, PG), 2022 Youth Fund of Natural Science Foundation of Hunan Province (2022JJ40215, PG), 2022 Supporting Programs of National Natural Science Foundation of China (BSJJ202101, PG), and Clinical Research center For Sports Medicine in Hunan Province, and the Key R&D Program of Hunan Province (2020SK2117, JW).

## Conflict of interest

The authors declare that the research was conducted in the absence of any commercial or financial relationships that could be construed as a potential conflict of interest.

## Publisher's note

All claims expressed in this article are solely those of the authors and do not necessarily represent those of their affiliated organizations, or those of the publisher, the editors and the reviewers. Any product that may be evaluated in this article, or claim that may be made by its manufacturer, is not guaranteed or endorsed by the publisher.
